# The Synthesis of Size-Adjustable Superparamagnetism Fe_3_O_4_ Hollow Microspheres

**DOI:** 10.1186/s11671-017-1986-z

**Published:** 2017-03-31

**Authors:** Chao Xu, Xiaolong Lu, Honglian Dai

**Affiliations:** 1 State Key Laboratory of Advanced Technology for Materials Synthesis and Processing, Wuhan University of Technology, Wuhan, 430070 People’s Republic of China; 2 Biomedical Materials and Engineering Research Center of Hubei Province, Wuhan University of Technology, Wuhan, 430070 People’s Republic of China

**Keywords:** Fe_3_O_4_ hollow microspheres, size-adjustable, Hydrothermal method, Superparamagnetism

## Abstract

One hundred fifty to 300-nm-sized monodisperse iron oxide (Fe_3_O_4_) hollow microspheres were synthesized by the one-pot hydrothermal method. The morphology and crystal structure of the as-prepared hollow microspheres was characterized by scanning electron microscopy, X-ray diffraction, transmission electron microscopy, and high-resolution transmission electron microscopy, while the magnetic property was investigated by vibrating sample magnetometer. We found that the particle size of the hollow microspheres was related to the amount of sodium citrate, polyacrylamide (PAM), and urea. The hollow structure of Fe_3_O_4_ microspheres has high magnetization saturation values ranging in 49.10–75.41 emu/g.

## Background

Hollow magnetic iron oxide (Fe_3_O_4_) microspheres have some characteristics, such as crystallinity, uniform sizes, biocompatibility, and surface area, and possess cavity and magnetic responsiveness [1]. These specific characteristics highlight Fe_3_O_4_ potential as a nanomaterial. There are many reports of preparing hollow porous magnetic Fe_3_O_4_ microspheres [2–10], and one-pot hydrothermal method is one of them [1, 11]. The advantages of the products obtained by this method are uniform sizes, crystallinity, and regular shapes [8]. Shanhu et al. [4] reported synthesizing of hollow nanospheres characterized by 290 nm in diameter and saturation magnetization reaching 83.0 emu/g. Lu-Ping et al. [12] obtained magnetite hollow spheres with an average diameter of about 310 nm and saturation magnetization 68 emu/g. However, they prepared solely single-size microspheres; there are few reports found about the synthesis of hollow Fe_3_O_4_ microspheres with adjustable size. Xuan et al. [13, 14] demonstrated the correlation between the size of the Fe_3_O_4_ nanoparticles and their magnetic properties. High saturation magnetization microsphere is sensitive to external magnetic field. Large microsphere cavities are able to hold many of guest molecules, which have potential to be used as drug carriers. Herein, we are reporting a one-pot hydrothermal method to fabricate size-adjustable Fe_3_O_4_ hollow microspheres.

In this work, a modified hydrothermal method [15] was developed to fabricate Fe_3_O_4_ microspheres. FeCl_3_·6H_2_O was used as the iron source; the size of the microspheres was adjusted by using different amounts of sodium citrate, urea, and polyacrylamide (PAM). All the Fe_3_O_4_ microsphere products are superparamagnetic and can form self-assembled secondary structure from the primary grains of the size about 18 nm. In addition, we synthesized hollow microspheres with sizes varying from 150 to 300 nm, the values of saturation magnetization were from 49.10 to 75.41 emu/g. The as-prepared Fe_3_O_4_ hollow microspheres have good hydrophilic, biocompatible, nontoxicity properties, and strong magnetic responsiveness, which allow them to serve as ideal candidates for practical applications such as magnetic resonance imaging, magnetic separation, and targeted drug delivery [16].

## Methods

### Materials

In our study, ferric chloride hexahydrate (FeCl_3_·6H_2_O, A.R., Sinopharm Chemical Reagent Co., Ltd) was used as iron source; urea (A.R., Shanghai chemical reagent co., Ltd) was used as alkali source. Polyacrylamide (PAM, Mn = 3,000,000) and trisodium sodium citrate were obtained from Sinopharm Chemical Reagent Co., Ltd. All chemicals were of analytical grade and used without further purification. The deionized water was prepared by UPT ultrapure water-polishing system.

### Preparation of Fe_3_O_4_ Hollow Microspheres

The superparamagnetism Fe_3_O_4_ hollow microspheres were prepared through a modified hydrothermal reaction [15]. Briefly, 2 mmol FeCl_3_·6H_2_O, 4 mmol sodium citrate, and 6 mmol urea were dissolved in 40 ml deionized water. Then, 0.3 g PAM was added under continuous stirring until it was dissolved totally and transferred into a Teflon-lined stainless steel autoclave (80-ml capacity). The autoclave was heated to 200 °C and maintained for 12 h, then it was cooled to room temperature. The black product was centrifuged and washed with deionized water and ethanol three times and then dried under vacuum overnight for further characterization.

### Synthesis of Size-Controllable Fe_3_O_4_ Hollow Microspheres

The above synthetic method can be extended to synthesize different diameter of superparamagnetism Fe_3_O_4_ hollow microsphere by varying the experiment parameters. Firstly, we changed the sodium citrate amount from 0 to 8 mmol without changing other parameters. Following, we increased urea amount while other conditions kept the same. Finally, we changed the PAM amount from 0.1 to 0.3 g to synthesize different sizes of microspheres. A series of experiments were carried out under different conditions as it has been summarized in Table 1.

**Table 1:** The detailed experimental conditions for the preparation of superparamagnetic Fe_3_O_4_ hollow microspheres

Sample	Sodium citrate	Urea	PAM	Time	Temperature
1	0 mmol	6 mmol	0.3 g	12 h	200 °C
2	2 mmol	6 mmol	0.3 g	12 h	200 °C
3	3 mmol	6 mmol	0.3 g	12 h	200 °C
4	4 mmol	6 mmol	0.3 g	12 h	200 °C
5	6 mmol	6 mmol	0.3 g	12 h	200 °C
6	8 mmol	6 mmol	0.3 g	12 h	200 °C
7	4 mmol	6 mmol	0.3 g	12 h	200 °C
8	4 mmol	8 mmol	0.3 g	12 h	200 °C
9	4 mmol	10 mmol	0.3 g	12 h	200 °C
10	4 mmol	15 mmol	0.3 g	12 h	200 °C
11	4 mmol	6 mmol	0.1 g	12 h	200 °C
12	4 mmol	6 mmol	0.2 g	12 h	200 °C
13	4 mmol	6 mmol	0.3 g	12 h	200 °C

Other conditions: FeCl_3_·6H_2_O 2 mmol, H_2_O 40 ml

### Sample Characterization

The phase structure of the samples was identified by powder X-ray diffraction (XRD) on a D8 Advance diffractometer using Cu Kα radiation (*λ* = 1.5418 Å) from 10° to 70° at a scanning speed of 4°/min^−1^. The morphology of the samples was observed using field emission scanning electron microscopy (FESEM, S-4800, Hitachi Corp, Japan) and high-resolution transmission electron microscopy (HRTEM, JEM-2100F STEM/EDS, JEOL Corp, Japan); all samples were microtomed to ultrathin sections for observation, using a LEICA ULTRACUT UCT. Fourier transform infrared (FT-IR) spectra were recorded on a Nicolet6700 (Nicolet, USA) spectrometer. The samples were dried and mixed with KBr to be compressed to a plate for measurement. Magnetic investigation was carried out at 300 K on a JDM-13 vibrating sample magnetometer.

## Results and Discussion

### Characterization of a Typical Sample

**Fig. 1:**
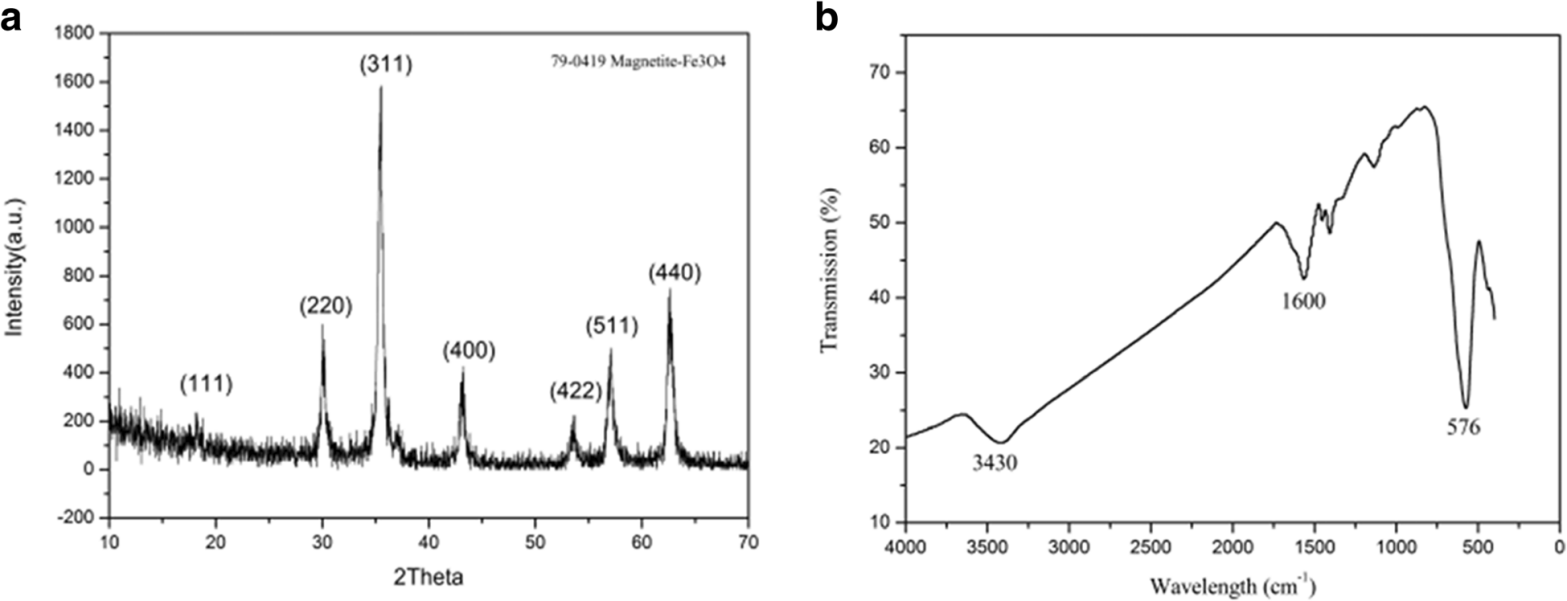
**a** XRD pattern of the typical sample. **b** FT-IR spectrum of the typical sample

Figure 1a shows the XRD pattern of sample synthesized at 200 °C. It was found that the intensities and *d* values of the peaks in the obtained XRD pattern match well with the Fe_3_O_4_ (JCPDS Card No. 79-0419). In addition, there are no impurity peaks found. We choose the [311] peak to calculate the average crystallite size of the sample according to the Scherrer formula, the result indicates an average crystallite size of 18.07 nm, then the sample went through a vibrating sample magnetometer measurement and showed superparamagnetic behaviors; this phenomenon was consistent with the report of Baoping et al that when the Fe_3_O_4_ nanoparticle diameters are smaller than 30 nm, they would exhibit superparamagnetic behaviors [17]. The FT-IR spectroscopy of Fe_3_O_4_ microspheres is shown in Fig. 1b. The peak at 576 cm^-1^ was the characterization of the Fe-O vibrations [18], the absorption peaks at 3430 and 1600 cm^-1^ was ascribed to –OH stretching vibration and bending vibration [11]. The results showed that the surface of the microspheres contains some hydrophilic groups, which may endow microspheres with hydrophilic properties.

**Fig. 2:**
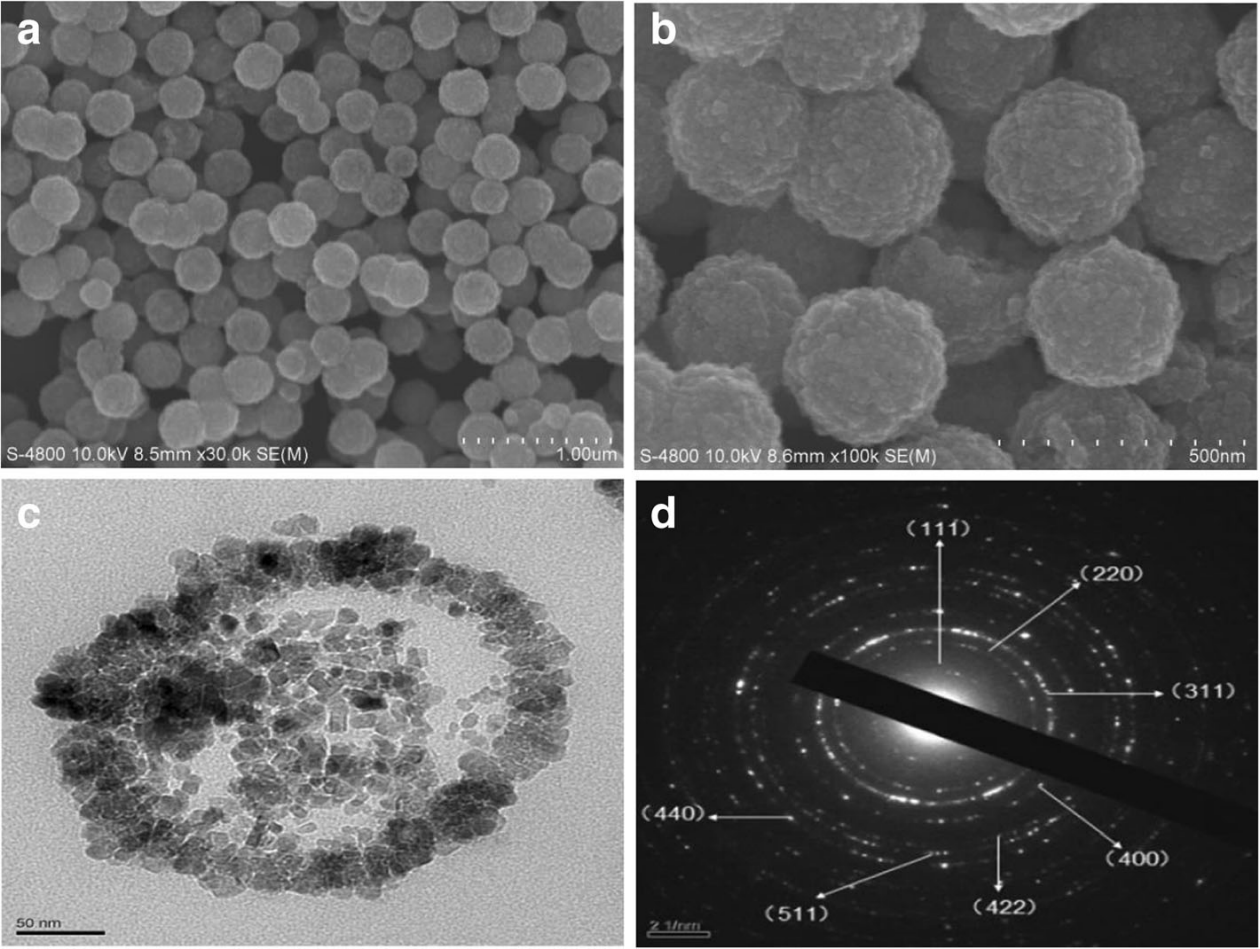
**a**, **b** SEM image of the typical sample. **c** TEM image of the typical sample. **d** SAED pattern from a single sphere

The morphology of the sample was characterized by SEM. The SEM images (Fig. 2a) showed that we have obtained uniform and monodisperse microspheres. The samples were cut in ultrathin sections and examined by TEM (Fig. 2b), which showed that the products were clusters of some small particles with coarse surfaces. The size of the hollow microspheres was about 300 nm in average. From the single microsphere TEM images (Fig. 2c), it could be found that the spheres had hollow internal structures. The corresponding SAED pattern taken from an individual microsphere is shown in Fig. 2d. It was found that the sample had polycrystalline structures, which were consistent with the TEM images that the microspheres consist of some small particles. From the inside to the outside, the rings can be indexed to (111), (220), (311), (400), (422), (511), and (440) planes of Fe_3_O_4_. All the diffraction rings can be readily indexed to the Fe_3_O_4_ phase.

### The Effect of Various Factors on Size-Controllable Synthesis of Fe_3_O_4_ Hollow Microspheres

#### The Effects of the Sodium Citrate on the Size of Product Particles

**Fig. 3:**
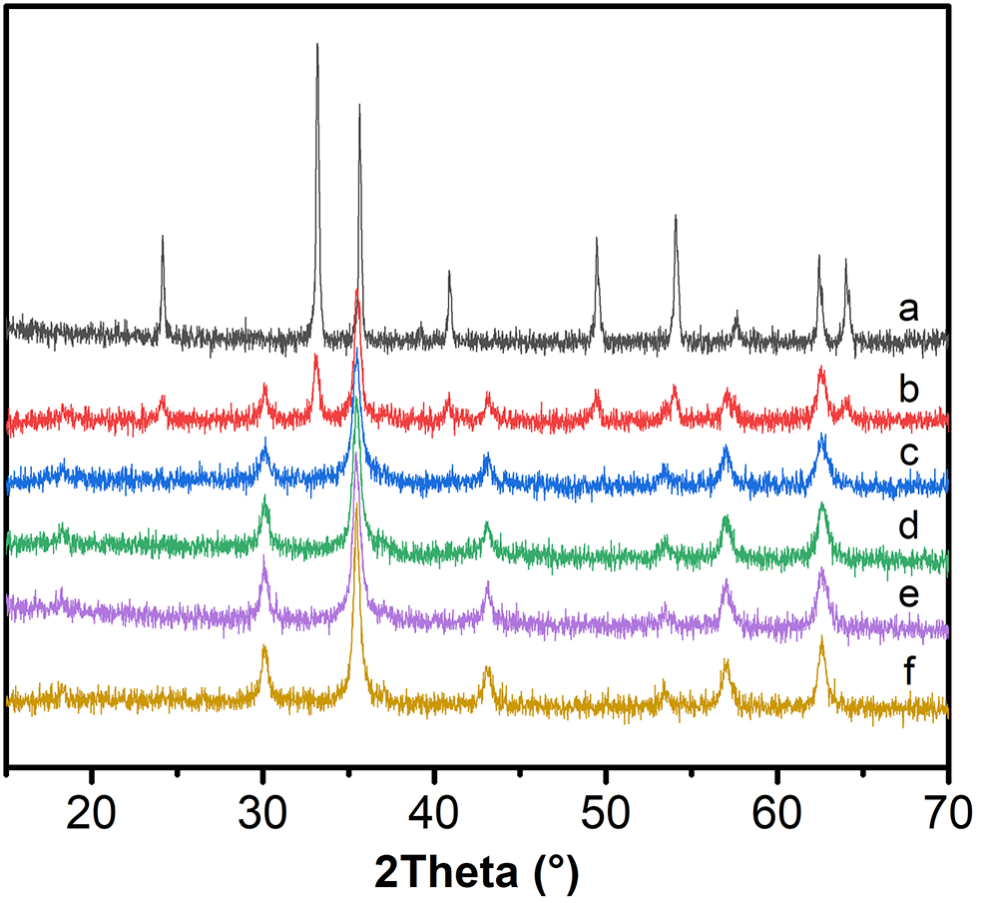
XRD pattern of different amount of trisodium citrate synthesized sample. *a* 0 mmol. *b* 2 mmol. *c* 3 mmol. *d* 4 mmol. *e* 6 mmol. *f* 8 mmol

The XRD patterns of the microspheres synthesized with different amounts of sodium citrate are shown in Fig. 3. α-Fe_2_O_3_ could be obtained when no sodium citrate is added (Fig. 3a), and the intensity of α-Fe_2_O_3_ gradually decreased with the increase of sodium citrate amount (Fig. 3b). When the amount of sodium citrate reach up to 3 mmol, the diffraction peaks of α-Fe_2_O_3_ disappeared completely. Sodium citrate seemed to play a role in the formation of the product. Sodium citrate might act as a reducing agent under high-temperature conditions [19, 20]. Furthermore, sodium citrate could also be used as a stabilizer in the system, every sodium citrate molecule containing three carboxyl groups, a part of carboxyl groups substituted Fe_3_O_4_ microspheres surface hydroxyl groups, and formed a monomolecular adsorption layer, which could reduce the reaction rate and inhibit grain growth [21].

**Fig. 4:**
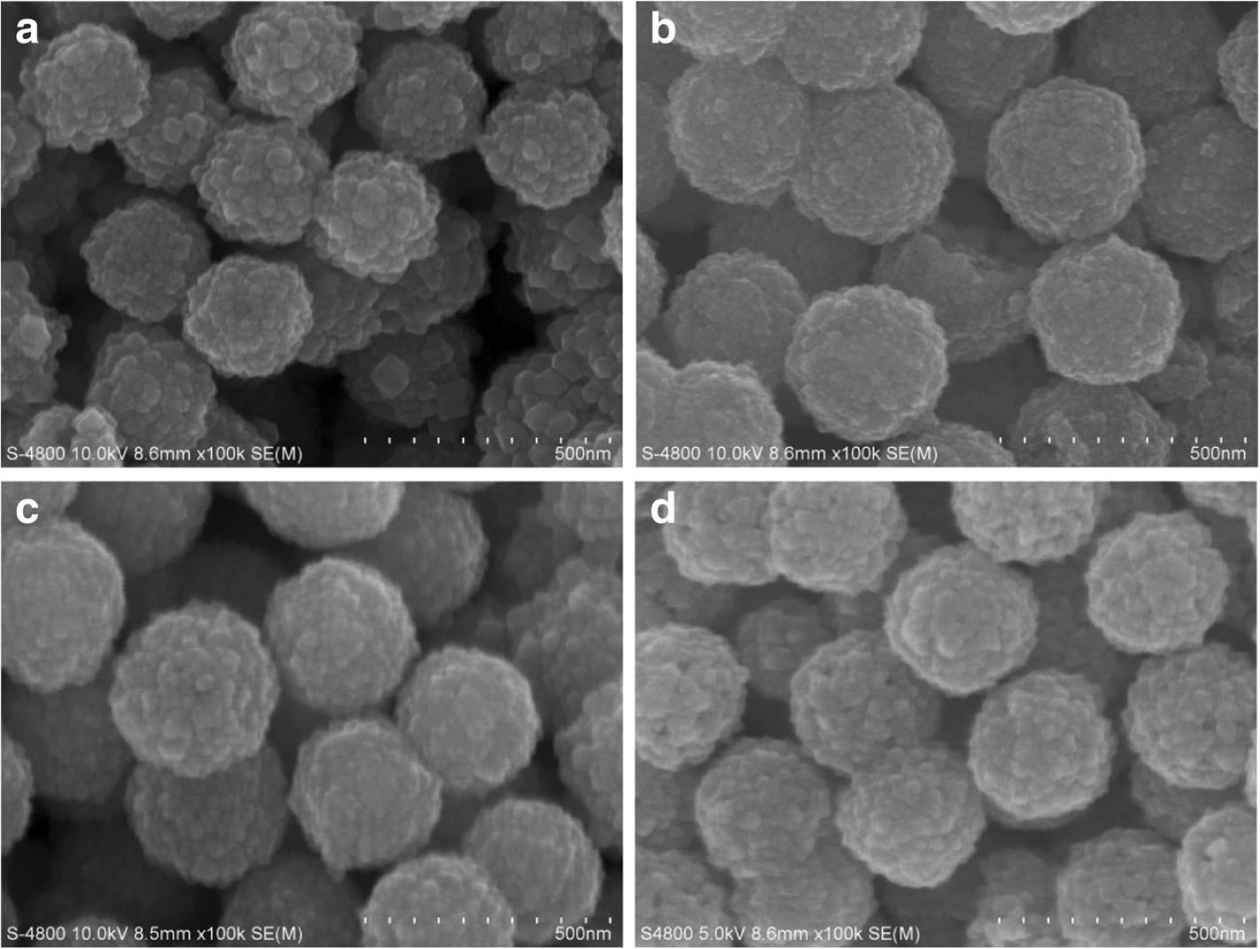
SEM images of different amount of trisodium citrate synthesized sample. **a** 3 mmol. **b** 4 mmol. **c** 6 mmol. **d** 8 mmol

From the XRD patterns, it could be found that when the amount of sodium citrate was 3, 4, 6, and 8 mmol, pure product could be prepared. The influence of the sodium citrate on the morphology of the products was examined by SEM. When the sodium citrate amount was 3 mmol, the diameters of the microspheres were about 250 nm (Fig. 4a). At the 4-mmol level, the sizes of the microspheres were 300 nm (Fig. 4b). Further increasing the amount up to 6 and 8 mmol, the sizes of the microspheres were still 300 nm, and no further morphology changes were found (Fig. 4c, d).

#### The Effects of the Urea on the Size of Product Particles

**Fig. 5:**
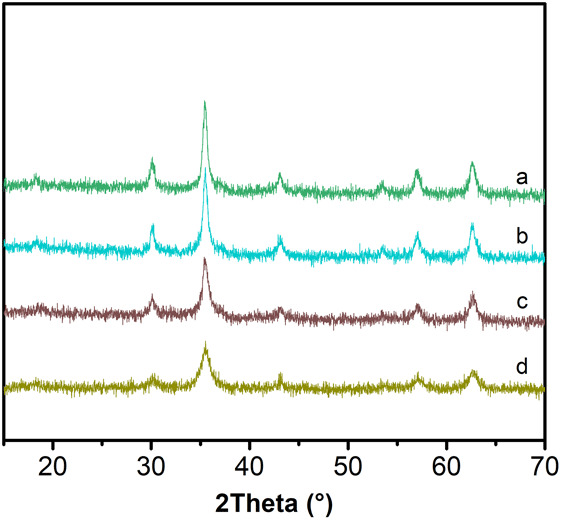
XRD pattern of different amount of urea synthesized sample. *a* 6 mmol. *b* 8 mmol. *c* 10 mmol. *d* 15 mmol

The influence of urea amount on the size of the Fe_3_O_4_ hollow microspheres was investigated through samples as listed in Table 1. The XRD patterns of the microspheres synthesized with different amounts of urea are shown in Fig. 5. All peaks of these four samples match well with standard Fe_3_O_4_ XRD diffraction (JCPDS Card No. 79-06419). No obvious impurity peaks are found in Fig. 5. The sharp peak indicated the high crystallinity of products. It indicated that urea as alkali source in the reaction system did not affect the formation of Fe_3_O_4_ crystal grains. In the reaction process, urea was decomposed to NH_3_ and provides an alkaline environment for the solution system [22].

**Fig. 6:**
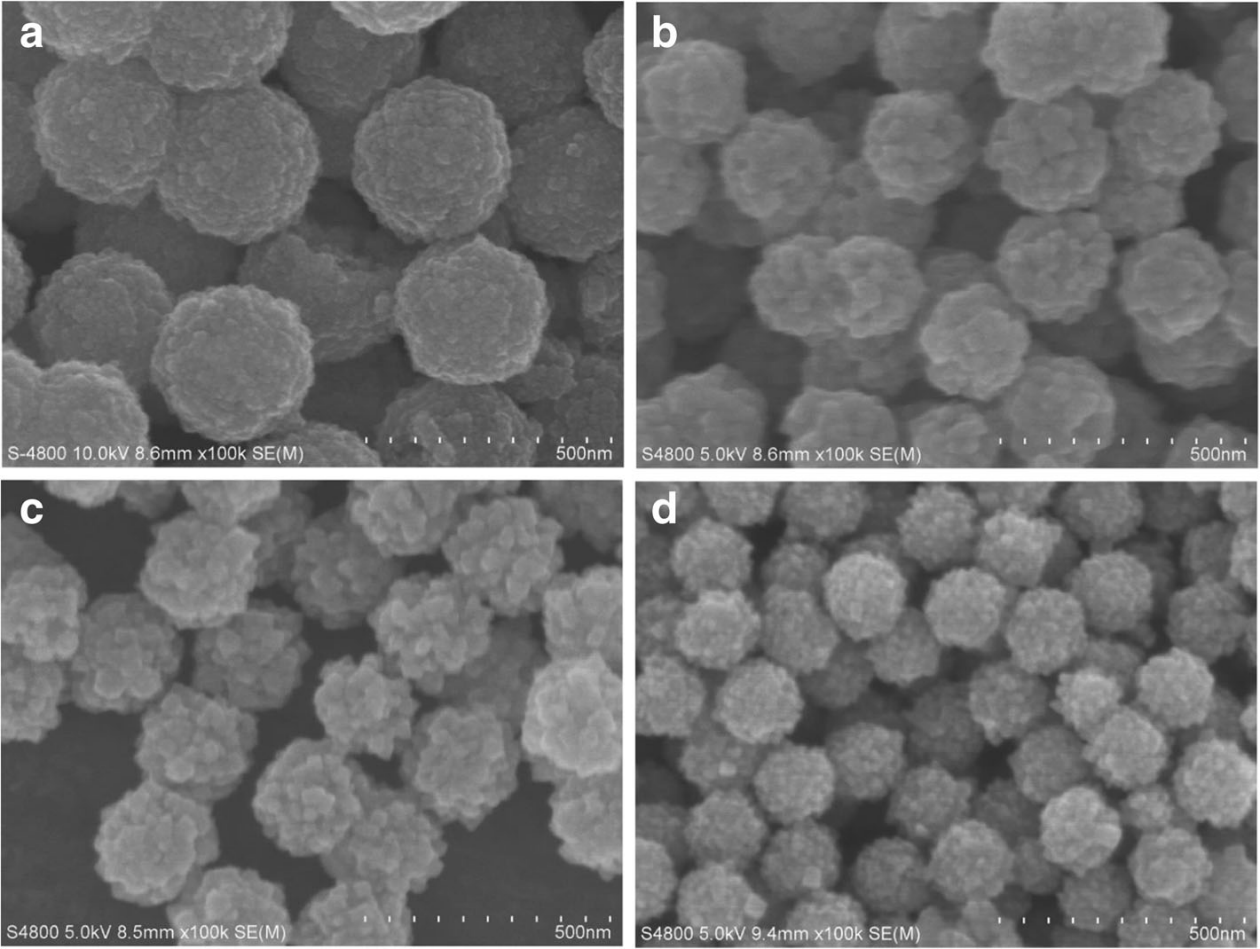
SEM images of different amount of urea synthesized sample. **a** 6 mmol. **b** 8 mmol. **c** 10 mmol. **d** 15 mmol

The morphology and size of the microspheres were examined by SEM. The SEM images showed that when the urea amount was 6 mmol, the diameters of the microspheres were about 300 nm (Fig. 6a). When the urea amount was increased to 8 mmol, the size of the microspheres decreased to 250 nm (Fig. 6b). Further increasing the urea amount to 10 mmol, the size of the microspheres decreased to 200 nm. When the urea amount reached up to 15 mmol, the size of the microspheres was 150 nm. It indicated that the amount of urea plays a role in the matter of the size of microspheres. However, with the increase of urea in the reaction, there were more NH_3_ and CO_2_ bubbles that act as a soft template [16], each soft template might adsorbed less nanoparticles; thus, smaller size of microspheres were obtained after Ostwald ripening process [23, 24].

#### The Effects of the PAM on the Size of Product Particles

**Fig. 7:**
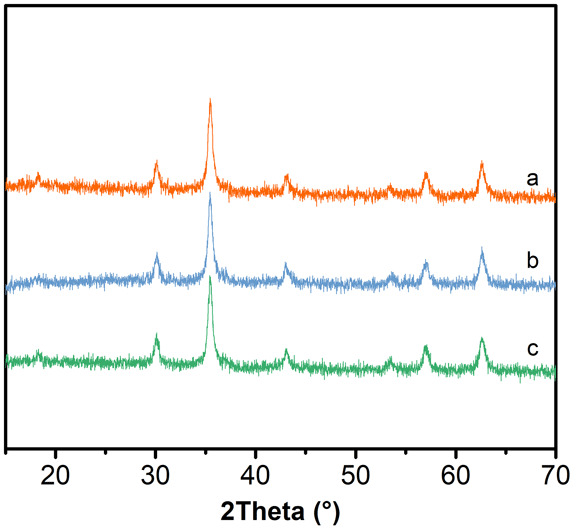
XRD pattern of different amount of PAM synthesized sample. *a* 0.1 g. *b* 0.2 g. *c* 0.3 g

**Fig. 8:**
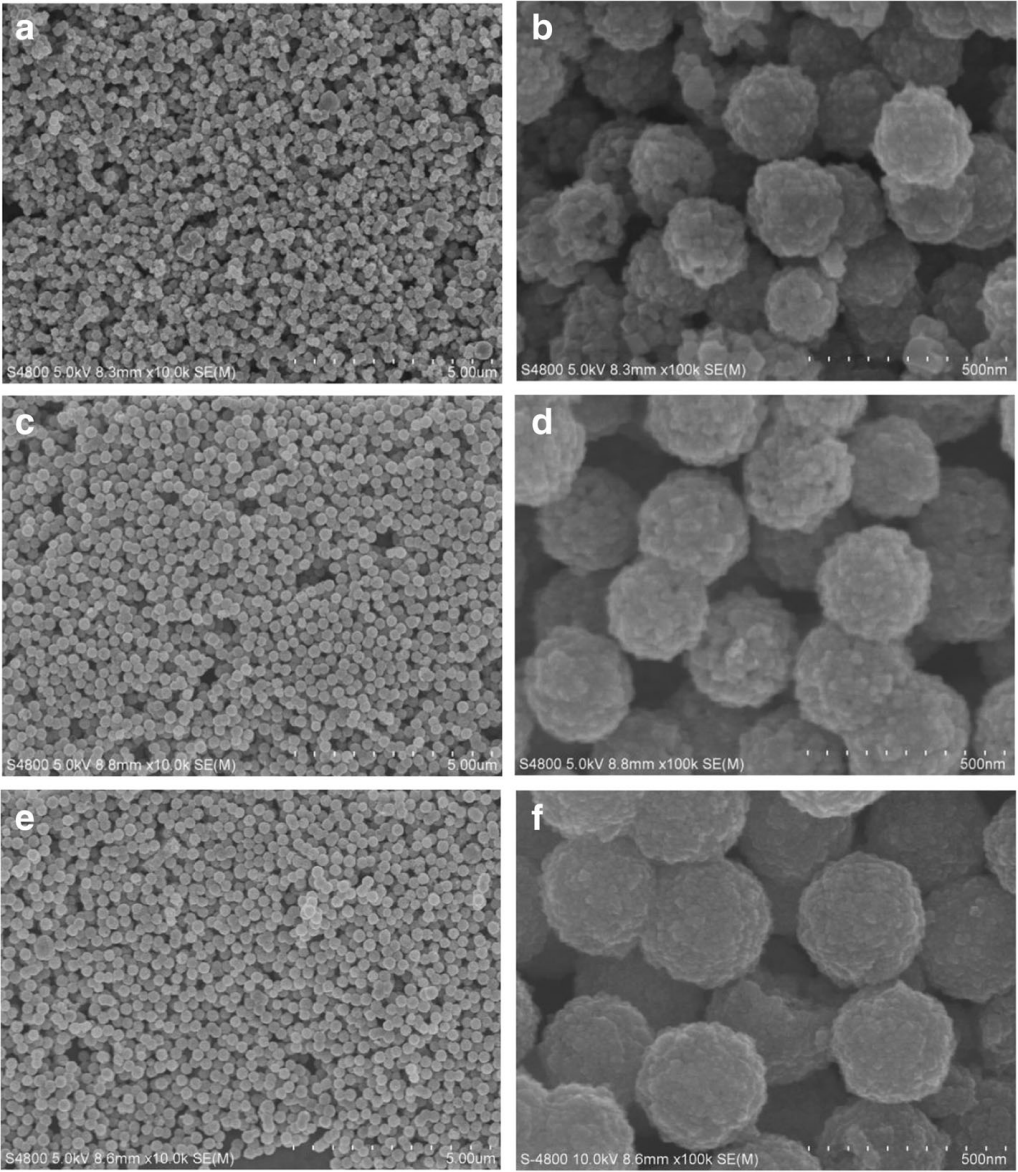
SEM images of different amount of PAM synthesized sample. **a**, **b** 0.1 g. **c**, **d** 0.2 g **e**, **f** 0.3 g

Figure 7 illustrates the XRD pattern of the samples as listed in Table 1. All peaks of these samples match well with standard Fe_3_O_4_ XRD diffraction (JCPDS Card No. 79-06419). No obvious impurity peaks were found. The sharp peak indicated that products had high crystallinity. The SEM images (Fig. 8) showed that when the PAM amount was 0.1 g, the sizes of the microspheres were about 200 nm (Fig. 8a, b). Dispersibility and shape of the samples were not good under such conditions. Increasing the amount up to 0.2 g, the dispersibility and shape of the samples were improved significantly, and the sizes of the microspheres increased to 250 nm (Fig. 8c, d). When the amount of PAM increased up to 0.3 g, the dispersibility and good shape of microspheres are obtained and the sizes of the microspheres were about 300 nm (Fig. 8e, f). Peng et al. reported that the polymer PAM contains a large number of amide ligands, consequently stabilizing the primary particles [25]. The polymer PAM might increase the viscosity of the solution, which might slow down the movement of nanoparticles, giving more time to adsorb the primary particles on the surface of soft templates and then self-assembled microspheres. With the increase of PAM concentration, nanoparticles had enough time to self-assemble into larger spheres.

### Crystal Structure and Magnetic Property of Fe_3_O_4_ hollow Microspheres with Different Sizes

**Fig. 9:**
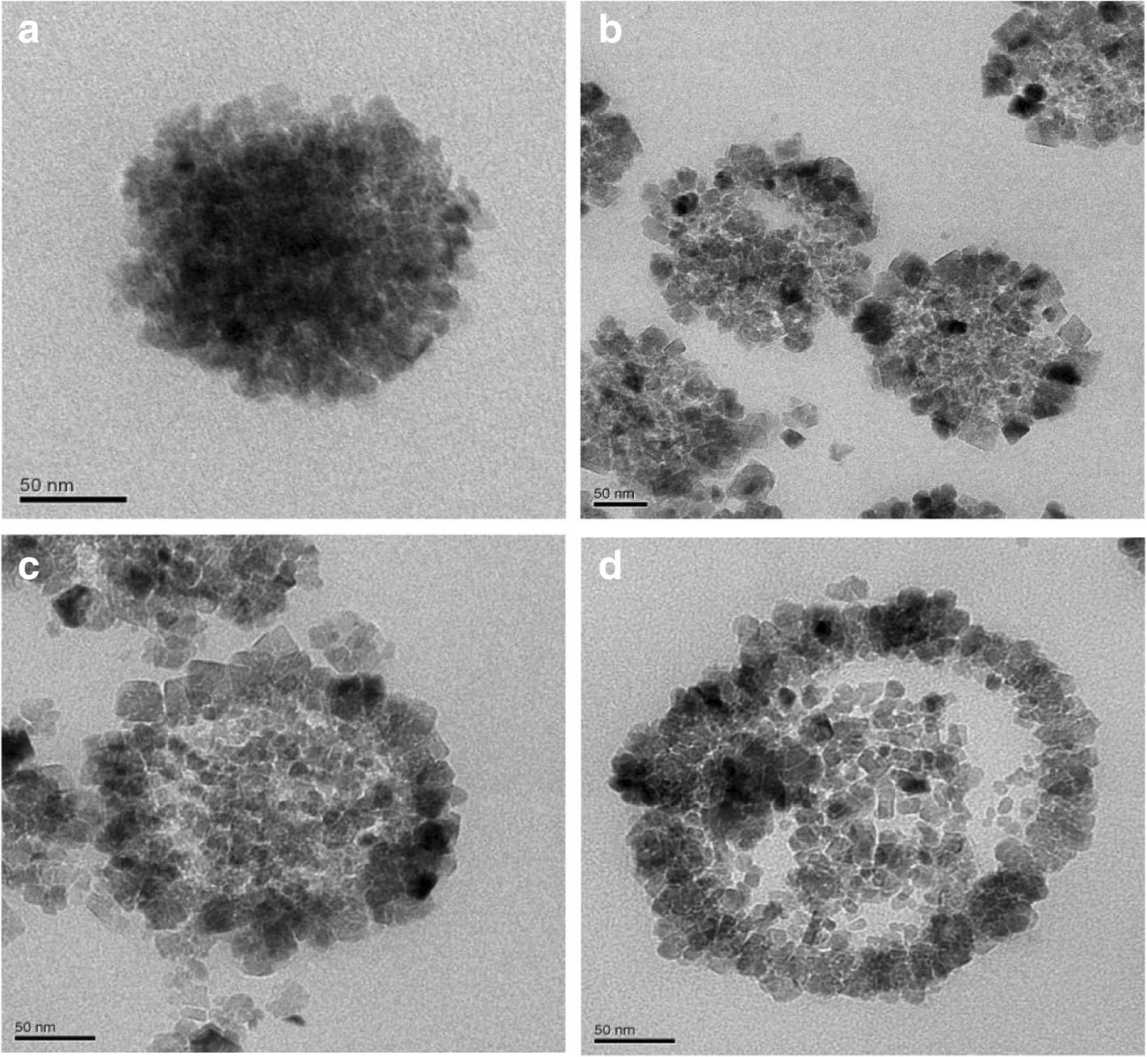
TEM images of Fe_3_O_4_ hollow microspheres at different sizes. **a** 150 nm. **b** 200 nm. **c** 250 nm. **d** 300 nm

We selected microspheres in the sizes of 150, 200, 250, and 300 nm and cut them in ultrathin sections and examined by TEM (Fig. 9). Fe_3_O_4_ microspheres with a diameter of 150 nm (Fig. 9a) were of solid structure. Fe_3_O_4_ microspheres with a diameter of about 200 nm (Fig. 9b) interior small nanoparticles were dissolved gradually. Fe_3_O_4_ microspheres with a diameter of 250 nm (Fig. 9c) were characterized as core-shell structure, and shell thickness were about 30 nm. Fe_3_O_4_ microspheres with a diameter of 300 nm (Fig. 9d) interior small nanoparticles were dissolved completely, which showed a significant hollow structure, and the out shell is composed of primary nanoparticles. Many cracks on the shell of the microspheres can be clearly observed, indicating the highly porous structure of the microspheres.

**Fig. 10:**
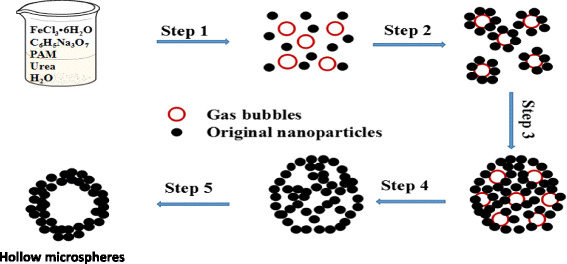
Schematic illustration of the formation mechanism of Fe_3_O_4_ hollow microspheres

Based on the experiment results and discussions above, we propose that the formation of the hollow spheres is a result of the dual role of gas bubbles and Ostwald ripening process. The formation mechanism is illustrated in a schematic diagram presented in Fig. 10. Urea decomposed into CO_2_ and NH_3_, which acted as soft templates in the reaction system (step 1). With the progress of the reaction, the original nanoparticles start to be adsorbed on the surface of the gas bubbles, owing to the high surface energy of the gas bubbles (step 2). Thereafter, the nanoparticles grew on the surface of the gas bubbles and agglomerated into loose spheres (step 3). Then, gas bubbles easily escaped from the loose spheres, which was also leading to form the hollow cavity (step 4) [26]. Nanocrystals located in the core region tend to dissolve owing to the higher surface energy than those nanocrystals on the outer surface, and the inner nanocrystals recrystallization on the outer shell attributes to the Ostwald ripening [7, 22]. Once the nanocrystals in the core are dissolved completely, a hollow cavity structure would form.

**Fig. 11:**
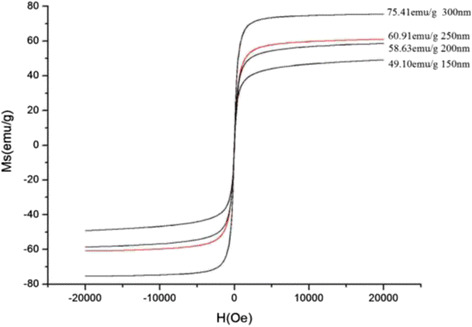
Room temperature magnetic hysteresis loops of Fe_3_O_4_ hollow microspheres at different sizes

Magnetic characterization of different sizes of microspheres measured at 300 K is shown in Fig. 11. The saturation magnetization values were 49.10, 58.63, 60.91, and 75.41 emu/g. The curves showed no remnant magnetization or coercivity; all microspheres exhibited superparamagnetic behavior at room temperature. The saturation magnetization values of prepared Fe_3_O_4_ microspheres increased gradually when particle size increased, which may be ascribed to the interior hollow cavity structure. The saturation magnetization values varied following the changes in sphere size, which allows our Fe_3_O_4_ microspheres to be controlled easily by an external magnetic field, which is favorable for their applications in the biomedicine field.

## Conclusions

A series of Fe_3_O_4_ hollow microspheres with a size of 150–300-nm particles were synthesized. The morphology and structure of the hollow Fe_3_O_4_ microspheres were studied by SEM, TEM, HRTEM, and XRD. We found that the size differences of Fe_3_O_4_ microspheres were related to the amounts of sodium citrate, polyacrylamide, and urea. The obtained Fe_3_O_4_ microspheres had a hollow structure and exhibited a superparamagnetic behavior with magnetization saturation values between 49.10 and 75.41 emu/g.

## Abbreviations

Fe_3_O_4_: Iron oxide
PAM: Polyacrylamide
